# Age and information preference: Neutral information sources in decision contexts

**DOI:** 10.1371/journal.pone.0268713

**Published:** 2022-07-18

**Authors:** Joshua L. Rutt, Derek M. Isaacowitz, Alexandra M. Freund

**Affiliations:** 1 Department of Psychology, University of Zurich, Zurich, Switzerland; 2 Department of Psychology, Northeastern University, Boston, Massachusetts, United States of America; 3 University Research Priority Program Dynamics of Healthy Aging, University of Zurich, Zurich, Switzerland; Temple University, UNITED STATES

## Abstract

Do adults of different ages differ in their focus on positive, negative, or neutral information when making decisions? Some research suggests an increasing preference for attending to and remembering positive over negative information with advancing age (i.e., an age-related positivity effect). However, these prior studies have largely neglected the potential role of neutral information. The current set of three studies used a multimethod approach, including self-reports (Study 1), eye tracking and choice among faces reflecting negative, neutral, or positive health-related (Study 2) and leisure-related information (Study 3). Gaze results from Studies 2 and 3 as well as self-reports from Study 1 showed a stronger preference for sources of neutral than for positive or negative information regardless of age. Findings also suggest a general preference for decision-relevant information from neutral compared to positive or negative sources. Focusing exclusively on the difference between positive (happy) and negative (angry) faces, results are in line with the age-related positivity effect (i.e., the difference in gaze duration between happy and angry faces was significantly larger for older than for younger adults). These findings underscore the importance of neutral information across age groups. Thus, most research on the positivity effect may be biased in that it does not consider the strong preference for neutral over positive information.

## Introduction

We are often confronted with decisions that require the weighing of the pros and cons of the different options. The best option is the one providing the most benefits and the fewest drawbacks. However, we often do not have the time or information processing capacity to gain and compare all information [1], necessitating us to focus on either on the benefits *or* the drawbacks. Given motivational changes across adulthood [2, 3] older and younger adults may differ in their tendency to focus on benefits (positive information) or on drawbacks (negative information) associated with different decision options.

According to a motivated cognition perspective [4], people are expected to place greater importance on information relevant to their goals—that is, positive information about options if the main goal is to obtain benefits, but negative information if the goal is primarily to avoid negative outcomes [5]. If no particular goal is active, people presumably choose to focus on information that contributes to positive mood, and show a greater preference for positive over neutral or negative information, as well as an avoidance of negative information [6].

### Value in positive information

According to Socioemotional Selectivity Theory (SST), older adults prioritize goals related to emotion regulation [3], and this motivation is reflected in their processing of information [7]. For example, older adults remember more positive than negative material [8], thereby potentially attempting to regulate their emotion in a positive direction [7]. Alternatively, older adults might already be in a more positive mood when processing the information compared to younger age groups [9], and remember more materials consistent with their mood [5, 10].

In the domain of visual processing, older adults appear to display a positive preference when they view emotional face pairs, looking toward positive and away from negative faces [11, 12]. Supporting the view that gaze patterns may be mood-regulating strategies, Isaacowitz and colleagues found that these preferences were activated when older adults were in negative moods [13], and that they may help older people to regulate how they feel [14].

These studies generally used passive viewing tasks in which participants were asked to look naturally, as if at home watching television. Such an instruction leaves open what the motivation of participants may be during the gaze task. It might be mood regulation [7] or it might be the search for informational value. Addressing this question, Depping and Freund [5] compared a condition in which participants read information on which they based a subsequent decision with a condition in which no decision was required. Using an incidental memory paradigm, they found that older adults remembered more negative information in the decision condition as compared to younger adults and to older adults in the no-decision condition.

Thus, during passive viewing, looking toward positive visual stimuli may be a good strategy when the goal is to increase or maintain positive mood. However, when viewing stimuli that contain decision-relevant information, attending more to negative information sources may be the better strategy when the primary goal is to avoid negative outcomes [5]. These gaze patterns may be markers of people’s motivational orientation and their goals. Gaze patterns might be more implicit and thus differ from people’s explicit self-reports.

### Value in negative information

Research on adult age differences in motivational orientation suggests a shift from a primary orientation towards gains and positive outcomes in young adulthood to an increasing importance of maintenance and avoiding losses or negative outcomes in older adulthood [15–19]. On the basis of this motivational shift, one would expect that younger adults mainly seek information that helps them to achieve positive outcomes, and that older adults mainly seek information that helps them to avoid negative outcomes or losses. Indeed, Depping and Freund [5] found in two experiments that, compared to younger adults, older participants remembered more negative than positive information in an incidental memory task for text-based scenarios requiring decisions between two options. Similarly, English and Carstensen [20] found no positivity effect when older adults made decisions that offered a chance to avoid negative outcomes; in contrast, age-related positivity effects were found for decisions that did not offer such a chance to avoid negative outcomes.

Consistent with the literature on age-related gaze patterns, and given the findings reported above, we expect older adults’ motivational orientation (toward avoiding losses) to manifest themselves in a gaze paradigms that require decisions, specifically decisions that offer a chance to avoid negative outcomes.

### Value in neutral information

People may place value on *receiving* positive information, or on *avoiding* negative information. However, it is impossible to distinguish between these two possibilities without also including neutral information. If people do not differ in their preference for neutral and positive information but prefer both over negative information, this would indicate an avoidance of negative information (but not a general seeking out of positive information). In contrast, if people prefer negative over positive and neutral information, they likely feel that negative valenced statements or images have higher informational value. By examining preference for neutral information sources (which, by definition, may be informative but is neither inherently positive nor negative in valence), along with any examination of positive or negative information preference, it is possible to quantify the extent that people actually *prefer* positive (or negative) information one the one hand as opposed to *avoiding* negative (or positive) information on the other hand.

Moreover, prior research suggests that information provided by neutral faces may have value in itself. Shlomo and colleagues [21] conducted two experiments in which people perceived neutral faces as more truthful than angry or ashamed faces. Additionally, in a series of experiments by Adams and colleagues [22], images of neutral older adult female faces predicted participants’ self-reported dispositional positive affect. These studies suggest the possibility that people may be influenced by neutral faces.

Bringing together the two lines of research on the positivity effect on the one hand and the shift of motivational orientation on the other, we expect people to prefer (choose, or attend more to) positive over negative information outside of decision contexts given that most people want to improve or maintain good mood. However, in decision contexts, we expect an age-differentiation based on the shift in motivational orientation. More specifically, we hypothesize that, due to their stronger orientation to avoid losses, older adults prefer negative over positive information so as to be able to avoid negative outcomes. In contrast, as younger adults’ goals are oriented primarily toward achieving gains rather than preventing losses or negative outcomes [15–18], younger adults should be less interested in negative information because it is less instrumental than positive information in order to optimize gains and positive outcomes. In addition, we hypothesize that neutral information will play an important role in these information preferences in that, when present, it may reflect a desire to avoid negative (or positive) information rather than to approach one or the other.

We addressed these questions by testing age differences in self-reported choice behavior and gaze patterns in a passive viewing task and contrasted it with a hypothetical decision task, using facial expressions as visual stimuli.

### The current studies

Study 1 investigated what kind of information people of different ages seek when gathering information as a basis for making a decision. Participants were asked to choose between hypothetical vacation packages. Before making the decision, they received information about the vacation packages based on information gathered, hypothetically, from people who recently experienced one of them. Participants were shown three sources of information as face images: a happy, a neutral, and an angry expression. The faces served as cues for positive, neutral, and negative information that could be obtained from the person in each of the photographs to inform the participant’s decision. Thus, Study 1 examined how people evaluate positive, negative, and neutral cues for information. In order to test if a preference for positive sources might be driven by the liking of friendly faces rather than the valence of the information itself, we compared faces versus text as the medium of information.

Including neutral information sources in addition to happy and angry sources, makes it possible to differentiate a *preference for* happy (or angry) stimuli from an *avoidance of* the opposite valence. Stated differently, including neutral information sources allows to answer the following question: do participants favor either happy or angry stimuli because it is the less aversive of the two options, or because they actually prefer it? In addition, as suggested by prior research, people may perceive a value on information coming from neutral facial expression cues; including neutral faces allowed us to test for this possibility.

In Studies 2 and 3, we sought to determine whether the self-reported preferences from Study 1 are also manifested in gaze patterns. Gaze studies use fixation patterns as a behavioral index of what visual stimuli participants find most goal-relevant in that context [6, 11]. Older adults’ self-reports of emotional processing may not be reflected in behavior [23]. We investigated this in two studies using different samples and different decisions (following Depping and Freund’s decision scenarios, Study 2 used a health-related decision, Study 3 a vacation-related decision). In each study, we introduced two different cues that we expected to differ in their perceived value as information sources. In Study 1, the two cues were faces and text. In Studies 2 and 3, the two types of cues we used were (i) faces that did not signal any kind of information just as they are typically used in passive viewing paradigms, and (ii) faces that served as cues for information about the hypothetical choice options. We used faces as cues not only because they are often used when studying the positivity effect, but also because evidence to date suggests that faces are a unique source of emotional information. For example, some evidence suggests that emotional face stimuli result in neural-level individual differences during information processing [24] and, in particular, during perceptual decision making about these stimuli. Moreover, facial expressions might illicit emotions in the viewer (e.g., happiness when seeing a happy face, fear when seeing an angry face). This is less likely when the information is provided in the form of text. Therefore, by including text as well in Study 1, we could consider how individuals utilize positive, negative and neutral information beyond the possibly unique context of face processing as well.

Across all studies, we compared positive, negative, and neutral information. Study 1 provides evaluative data reflecting participants’ self-reported reasons for why they might value certain types of information over others. Gaze patterns in Studies 2 and 3 indicates to which type of information sources they attend most. Finally, choice data from Studies 2 and 3 reflect which types of cues they ultimately choose as sources of information.

## Study 1

To examine how the valence of an information source influences people’s preference for the information, and whether this differs by age, Study 1 assessed self-reports in the context of hypothetical decision scenarios: deciding among travel packages for a vacation and deciding among allergy remedies. We chose vacations and allergy medicines because all age groups are similarly likely to go on vacations and to be affected by allergies. With this approach, we aimed to hold the age-relevance of the hypothetical decisions constant. We hypothesized that older adults prefer information provided by the negative (angry) information source, reflecting a motivation to avoid or minimize potential losses, but younger adults the information provided by the positive information source, reflecting a motivation to maximize potential gains.

Information about each decision option was offered in the form of positive, negative and neutral feedback regarding other people’s personal experiences with the various options. Separate feedback was offered for multiple aspects of the decision options. In Study 1, we investigated how favorably people making a decision evaluate potential sources of information depending on the valence. That is: How favorably (and for what reasons) do people perceive positively or negatively valenced, or neutral information about one of their decision options? Specifically, participants in the current study rated the helpfulness, trustworthiness, and informational value of each of the facial expressions. We consider “neutral” stimuli as lacking any observable cues to valence. In practice this meant that we selected facial expressions that had been rated by a sample of younger, middle-aged, and older adults as neutral in the FACES database [25]. Neutral facial expressions generally lack the muscle movements associated with different positive or negative emotion expressions [26].

Photographs of a positive (happy), neutral, and negative (angry) emotional expression of the same person were shown simultaneously on the screen. The three emotional expressions represented positive, neutral, or negative information about a particular decision option, that would be provided by the person in the picture (i.e., information about the decision option that made the person happy, neutral, or angry). We also assessed these same ratings without photographs depicting emotional expressions faces by providing the same positive, neutral, and negative information on the screen in the form of text (i.e., participants saw written information). This allowed us to test the potential influence of the information *source* (i.e., emotional faces versus text). This is especially important given prior findings regarding neutral faces in particular—for example, perceiving them as more truthful than emotional faces [21]. We investigated these aspects of information preference for two separate hypothetical scenarios, a health-related decision on allergy medicine that is typically geared at avoiding a negative state (i.e., suffering from allergies), and a leisure-related decision on vacation packages that is typically geared at optimizing a positive state (i.e., enjoyment of the vacation).

### Method

#### Participants

The sample (*N* = 182) was recruited using the research participant pool of the Life-Management Laboratory at the University of Zurich (*n* = 64) and an online-recruitment firm (Respondi.de; *n* = 118), targeting adults between 18 and 80+ years in German-speaking Switzerland. We aimed for sufficient numbers of young, middle-aged, and older adults to be able to detect differences in information preference by age group, valence, and information source condition. As recommended for complex designs such as ours with more than two factors [27], we used a Monte Carlo simulation method based on our expected pattern of means, standard deviations, and correlation among repeated measures. We estimated small mean differences across cells, allowing for an interaction across the three factors (i.e., different positive-negative valence patterns for faces as compared to text, with this effect depending on age) along with logical standard deviations (equal for all cells; *SD* = 1.5), and a correlation of 0.30 among repeated measures. Our alpha was 0.05, and our predicted sample size (which was feasible based on prior studies in the lab) was *N* = 180 participants (60 in each age category). A series of 2000 simulations produced hypothetical data to estimate that our power to detect a significant 3-way interaction among the three factors (Holm adjustment for multiple comparisons). Our power was 0.87 to be able to detect an effect size (Partial Eta Squared) of 0.05 for the 3-way interaction. A major advantage to estimating sample size using this method is that we specify expected results in terms of means and standard deviations, rather than effect size.

The sample consisted of *n* = 61 younger (18–30 years, *M* = 24.13, *SD* = 3.83, 60.7% female), *n* = 61 middle-aged (41–55 years, *M* = 48.58 years, *SD* = 4.12, 60.7% female), and *n* = 60 older adults (65–81 years, *M* = 72.58, *SD* = 3.83, 51.7% female). Table 1 shows descriptive sample characteristics by age group. The recruitment company has a fixed reimbursement plan, which resulted in a compensation of participants with EUR 3 (equivalent to 3 USD). For participants recruited through the participant pool of the Life-Management Laboratory at the University of Zurich, a contribution of 4 Swiss francs was made on their behalf to the charity organization Doctors Without Borders.

**Table 1:** Participant characteristics Study 1.

	Younger		Middle-Aged		Older		
Measure	*M*	*SD*	*M*	*SD*	*M*	*SD*	*F*(2, 179)	*p*
Overall life satisfaction	5.03	1.25	5.28	1.16	5.75	0.91	6.43	.002
Overall physical health	5.39	1.01	5.34	0.93	5.40	0.98	0.06	*ns*
MDBF								
Pretest positive	3.49	1.07	3.65	1.14	4.29	1.00	9.45	< .001
Pretest negative	2.86	1.05	2.22	1.40	1.66	1.01	15.99	< .001
Posttest positive	3.41	1.10	3.77	0.99	4.30	0.96	11.64	< .001
Posttest negative	2.40	1.16	1.83	1.14	1.30	1.01	15.09	< .001
	*%*		*%*		*%*		*χ* ^2^		*p*
Education							52.59	< .001
Obligatory school	13.1		3.3		5.0			
Apprenticeship	27.9		44.3		28.3			
Upper professional training	8.2		16.4		31.7			
High school	34.4		4.9		6.7			
2-year college	1.6		4.9		16.7			
University degree	14.8		26.2		11.7			
Family status								
At least one child	4.9		59.0		83.3		78.19	< .001
In a stable relationship	31.1		54.1		61.7		12.27	.002

*Note*. MDBF = Multidimensional Mood Questionnaire. The MDBF measures were assessed before and after the main experimental task separately for positive and negative mood.

#### Design

The experiment had a 3 (age; younger, middle-aged, older) x 3 (valence; happy, neutral, angry) x 2 (condition; faces, text) mixed design (age as between-subjects; valence and condition as within-subjects). All response scales used a 7-point Likert scale ranging from 0 to 6. The two domains, health and vacation, were analyzed separately because we were not interested in domain as a within-subject factor; rather, we included two domains to test if the findings generalize across domains that are typically geared towards avoiding a negative state or optimizing a positive state.

#### Socio-demographics

Participants reported on socio-demographic characteristics including age, education level, and family status. Participants reported subjective health (“All in all, how do you evaluate your physical health?”) and life satisfaction (“All in all, how satisfied are you with your life?”). Results are provided in Table 1.

#### Mood

Participants completed a short version of the Multidimensional Mood Questionnaire [28] both before the main task (short form A) and after the main task (short form B). We averaged scores from the 6 positive and the 6 negative items to obtain a single score for positive mood and a single score for negative mood (see Table 1). This assessment allowed us to test if baseline mood influenced participants’ ratings of positive and negative information. In addition, pre- and post-test mood scores allowed us to assess whether ratings toward positive (or away from negative) stimuli were associated with subsequent mood changes.

#### Information preference

A hypothetical decision scenario was used to measure information preference, presented as blocked questions for two decision scenarios, one involving a decision among vacation packages, and the other a decision among allergy remedies. The order of the two decision scenario blocks was counterbalanced across participants. For the vacation package scenario, participants decided from three different travel package options; for the health scenario, participants decided among three allergy medicines. For both decision scenarios, each of the two information sources (faces vs. text) was presented as a separate block of six trials (two trials for each of the three travel packages / allergy medicines).

**Fig 1:**
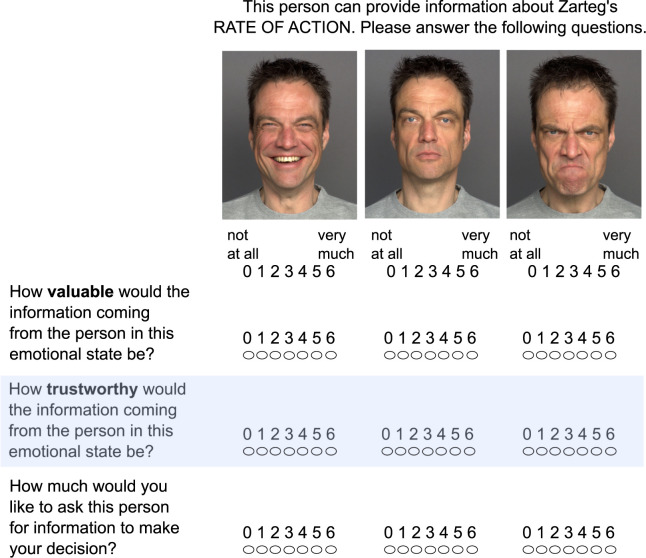
Example stimulus screen; faces condition (i.e., participants see face images but do not see the actual information); health scenario. Examples of information displayed in the text condition are shown in Table 2.

For the “faces” condition, each trial displayed a separate screen that provided information on one of the three travel options. Each trial presented three face images representing a person who had hypothetically tried that particular option and could offer feedback. Each face image expressed a different emotion (happy, neutral, angry), which represented the type of information (positive, neutral, negative) that the person could provide about a specific attribute of the option presented. Information about a different attribute was provided for each of the six trials, thus each trial presented information on one of six possible attributes (i.e., landscape, beach, food, general service, weather, and costs). As an example, one trial presented information about the quality of the food of one of the vacation packages, and evaluations of the food could be obtained from one of three facial expression images—happy, neutral, or angry—each representing the type of information the person would provide (see S1 Appendix for the specific attributes and decision options for the six “faces” condition trials). Faces were color photographs from the FACES Database [25]; see Fig 1. For this “faces” condition, no actual information about the decision options was given. Rather, participants had to select the information *source* (i.e., a person with a happy, neutral, or angry facial expression) from which they would prefer to obtain information.

For each of the three face images, participants rated: 1. How helpful the information would be coming from the person in this emotional state; 2. How trustworthy the information would be from the person in this emotional state; and 3. How much they would like to ask the person in this emotional state to give them the information. Participants then continued to the next trial and provided these same ratings but in response to a different person’s three face images who offered information on another attribute of one of the decision options. The six people whose face images were used for the travel package scenario were different from the six used for the allergy remedy scenario. Participants saw only the faces for this condition but did not see the printed positive, neutral, and negative information.

For the “text” condition, each of the six trials presented text about one of the options. Participants were told again that the information came from people who had hypothetically tried one of the three options and could offer feedback. Each piece of information provided either positive, neutral, or negative information about the decision option in the form of text. For this condition, no images depicting emotional states were shown. The three pieces of information were printed in the same position on the screen as in the “faces” condition. Unlike the “faces” condition, however, participants rated printed information about the decision option that was provided on screen rather than rating facial expression images as cues for the information they *could* obtain. Examples of the actual positive, neutral, and negative text information (on which participants based their ratings) is shown in Table 2. As in the “faces” condition, participants rated for each of the three pieces of information how (1) *helpful*, (2) *trustworthy*, (3) *sought out* the information would be coming from this source. They provided these same ratings for each of the six trials (each offering information about a different attribute of one of their travel package options or allergy medication, respectively). For each of the two decision scenarios, all participants completed the “faces” condition first, followed by the “text” condition.

**Table 2:** Positive, neutral, and negative information examples Study 1 and Study 2.

Criteria	Statement	Valence
Doctor Recommendations	My family doctor recommended Zarteg because of its high potency.	Positive
Speed of Action	The delicate blades helped me within a very short time.	Positive
Cost	I have health insurance.	Neutral
Side Effects	Possible side effects are listed on the package leaflet.	Neutral
Duration of Action	The Zarteg tablets helped only briefly.	Negative
Long-Term Effectiveness	Shortly after I stopped taking Zarteg, the allergy returned.	Negative

The allergy remedy scenario was structured in the same way as the vacation package scenario.

#### Procedure

The study was completed online using SoSci Survey [29]. Participants provided informed consent, then completed demographic questions and a mood questionnaire. They then completed one of the two decision scenarios (health or vacation), and then completed the other decision scenario. The order of the two decision scenarios was counterbalanced across participants. For each scenario, participants began with the “faces” block. They were shown a screen that indicated a particular attribute about one of their decision options (an example is shown in Fig 1; additional examples are shown in S1 Appendix). The screen also displayed three face images (a happy, neutral, and angry face) of the same person (see Fig 1). Next, on the same screen, participants provided their ratings to our three questions (see Fig 1). After providing the three ratings, the next trial concerning a different attribute of one of the decision options. After six such trials, the “text” block began that followed the same procedure (see Fig 1). Next, they completed the post-test mood questionnaire. Participants were then thanked and debriefed. The entire session lasted approximately 30 minutes.

The research was carried out in accordance with the regulations of the Ethics Committee of the Faculty of Arts and Sciences at the University of Zurich. These regulations define a two-stage process of ethical clearance, the first stage of which is a self-assessment of ethical risks by the researcher according to a checklist provided by the Ethics Committee. The present research passed the first stage, and therefore was exempt from further review by the Ethics Committee.

### Results

#### Information preference in decision scenarios

Ratings of helpfulness, trustworthiness, and informational value of the source were positively correlated for both faces and text, and within both domains (*r*s > .33; *p*s < .01). Therefore, we aggregated these three ratings into a single *information value score* which we used as our dependent variable for the following analyses. We generated this score separately for faces and information, and also separately by health and vacation domain. (Results were unchanged when helpfulness, trustworthiness, and informational value were analyzed separately; see S1 File).

Age differences in how valuable the participants rated the information that could be obtained from the three information sources (happy, neutral, angry) in both conditions (i.e., faces and text) were examined separately for each domain (health and vacation) in a 3 (age: younger, middle-aged, older) x 3 (valence: happy, neutral, angry) x 2 (condition: faces, text) repeated measures ANOVA with age group as the between-subject factor and the other two as within-subjects factors. Whenever appropriate, Greenhouse-Geisser correction was applied to the degrees of freedom to account for deviations from sphericity.

**Fig 2:**
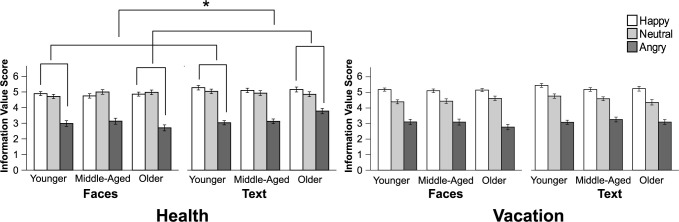
Mean ratings information value score; by condition (faces, text) and age (health = left, vacation = right). Younger = 18–30 years; Middle-Aged = 41–55 years; Older = 65–81 years. Error bars indicate SEM. * *p* < .01.

For the health domain, results revealed main effects for valence, *F*(1.64, 292.92) = 386.06, *p* < .001, η_p_^2^ = .68; and condition, *F*(1, 179) = 13.24, *p* < .001, η_p_^2^ = .07. These main effects were qualified by a valence x condition interaction, *F*(1.54, 275.86) = 3.80, *p =* .034, η_p_^2^ = .02. This lower-order interaction was qualified by an age x valence x condition interaction, *F*(3.08, 280.90) = 6.67, *p* < .001, η_p_^2^ = .07. We investigated the three-way interaction further by testing for age differences (older vs. younger adults), by condition (faces vs. text), and valence (positive vs. negative) in information value score. Both age groups rated negative sources as less valuable than positive sources. However, older adults showed a greater aversion to negative relative to positive sources in the faces condition than in text condition, and this difference was larger for older adults (mean difference = 0.77) than for younger adults (mean difference = -0.33), *t*(179) = 3.11, *p* = .002, *d* = 0.23 (see Fig 2). Because neutral information sources were rated notably high in the faces condition, we also tested whether older and younger adults differed by condition with regard to negative compared to neutral ratings. Older adults showed a greater aversion to negative relative to neutral sources in the faces condition than in the text condition, and this difference was larger for older adults (mean difference = 1.20) than for younger adults (mean difference = - 0.28), *t*(179) = 3.92, *p* = .001, *d* = 0.29. (See S1 File for additional means and standard deviations).

For the vacation domain, results revealed main effects for valence, *F*(1.66, 296.26) = 435.63, *p* < .001, η_p_^2^ = .71; and condition, *F*(1, 179) = 4.10, *p* < .05, η_p_^2^ = .02. No other main effects or interactions were significant. As can be seen in Fig 2, results were generally in the same direction as for the health domain, but not significant.

#### Mood

Both pre- and post-test mood scores differed by age group (*p*s < .001) in that older compared to younger adults reported more positive and the less negative mood (see Table 1). Higher positive mood (both pre- and post-test) significantly correlated with higher average ratings for positive sources and, albeit to a lesser degree, with higher average ratings for negative sources. Pre-test positive mood correlated with average positive rating (*p* >.01) and pre-test negative mood correlated negatively with average negative rating (*p* < .05), suggesting that participants seek out information that is congruent with their mood. Neither average positive nor negative information source ratings correlated significantly with a change in mood in the post-test (see Table 3). In other words: the main ratings task did not influence mood significantly, suggesting that rating tendencies did not serve as a mood-regulating strategy.

**Table 3:** Correlations between mood before and after the information preference task and average positive and negative ratings.

Measure	1	2	3	4	5	6	7	8
1. MDBF pretest positive	—	-.60**	.60**	-.50**	-.49**	.16*	.20**	.18*
2. MDBF pretest negative	-.60**	—	-.50**	.62**	.14	-.49**	-.06	-.15*
3. MDBF posttest positive	.60**	-.50**	—	-.79**	.41**	-.29**	.27**	.18*
4. MDBF posttest negative	-.50**	.62**	-.79**	—	-.29**	.38**	-.14	-.14
5. Mood change positive	-.49**	.14	.41**	-.29**	—	-.50**	.07	-.01
6. Mood change negative	.16*	-.49**	-.29**	.38**	-.50**	—	-.08	.02
7. Average positive rating	.20**	-.06	.27**	-.14	.07	-.08	—	.21**
8. Average negative rating	.18*	-.15*	.18*	-.14	-.01	.02	.21**	—

*Note*. MDBF = Multidimensional Mood Questionnaire. The MDBF measures were assessed before and after the main experimental task separately for positive and negative mood. Mood change = posttest–pretest.

* *p* < .05.

** *p* < .01.

### Discussion

Results of Study 1 suggest that adults of all ages place considerable value on health-related information obtained from people who are in either a neutral or a positive emotional state, but very little value on information from people in an angry emotional state. The strong rating for neutral relative to negative, particularly in older adults, did not extend to text sources where participants of all ages preferred positive over both neutral and negative information. In line with differences between a health- and a vacation-related scenario found by Depping and Freund [5], the vacation scenario generally revealed a positive preference for both faces and text sources.

Additionally, older adults evaluated angry emotional faces not to be a valuable information source. Although all age groups rated angry faces less favorably than other faces, this pattern was most pronounced in older adults. Regarding negative text, all age groups rated negative information to be of lower value to gather information about the decision options than positive or neutral information, but this pattern was *least* pronounced in older adults (for ratings of health information).

Importantly, the stronger-than-expected preference for neutral face stimuli in this study suggests that people may avoid angry sources for information, and value both neutral and happy sources as more reliable or informative. In fact, it might be the case that people associate anger with being the worst source for information because anger is a strong emotion that might be perceived as clouding people’s judgments. In line with theorizing by Charles and colleagues [8] that highly arousing negative emotions are particularly averse to older adults, this may be especially relevant to older adults—who rated angry faces to be of much lower value as a source of health information than happy or neutral other faces. Had our study included only happy and angry faces, older adults’ choices may have resembled a preference for positive rather than an avoidance of negative.

To further understand these age-related decision-related information preference patterns, Studies 2 and 3 focused on different methods to assess the construct of preference. Study 1 measured people’s explicit self-reported preferences for certain sources of information, and Studies 2 and 3 examined if these preferences are also reflected in the gaze patterns using the same stimuli and scenarios.

Building on the studies by Depping and Freund [5] that found significant differences between information processing when a task requires a decision versus not, we again included both conditions: the first required no decision; the second did require a decision (passive viewing vs. decision task, respectively). In a passive viewing paradigm, utilizing and/or looking toward positive faces may be a good strategy when the goal is to increase or maintain positive mood. However, when the visual stimuli contain decision-relevant information, utilizing and/or attending more to negative information may be the better strategy when the primary goal is to avoid negative outcomes [5], and more to positive information when the goal is to achieve positive outcomes.

Health and vacation scenarios were examined separately in Studies 2 and 3, respectively. We hypothesized that older adults prefer happy faces over angry faces for passive viewing conditions, but angry faces over happy faces for decision task conditions in order to inform decisions that will avoid negative outcomes. We expected younger adults to show a preference for happy faces across both passive viewing and decision task conditions in order to optimize positive outcomes.

## Study 2

Using a decision task, this study assessed gaze patterns as well as behavioral preferences of young, middle-aged, and older adults regarding information about four different allergy medicines. As in Study 1, participants were shown simultaneously three images of the same person with three different facial expressions (happy, neutral, angry; from left to right). Of particular interest were age-differences in gaze patterns between their first viewing of the faces without having to make a decision and their second, decision-related viewing. In the second viewing, participants selected one of the faces on each trial to provide information about one of their allergy remedy options so that they could make an informed decision. Thus, gaze patterns during the decision task reflect gaze toward faces as cues for potentially instrumental information. Gaze patterns during passive viewing reflect gaze toward a face image with no specific instrumental information. Differences between conditions thus reflect the effect of gaze changes when faces serve as cues for information relative to when they do not signal specific information about the choice options. Assessing gaze represents an unobtrusive way to measure preferences beyond self-reports (which might be more biased by conforming to social desirability). Again, including both neutral and valenced faces allowed us to test our hypotheses regarding age and positive versus negative outcomes as well as the potential value in neutral faces especially when they serve as source of decision-relevant information.

### Method

#### Participants

Participants (*N* = 181) were recruited from participant pool of the Life-Management Laboratory at the University of Zurich, senior clubs and fitness centers, and flyers. Due to technical problems or difficulties with calibration, eye movements were not trackable for 12 participants (7%), in at least 25% of the trials, resulting in a final sample size of *N* = 169. The sample contained *n* = 58 younger adults (18–30 years, *M* = 22.88, *SD* = 2.78, 58.6% female), *n* = 55 middle aged adults (40–55 years, *M* = 46.71, *SD* = 4.66, 69.1% female), and *n* = 56 older adults (65–85 years, *M* = 71.50, *SD* = 4.85, 50.0% female). Using a similar method as Study 1, we based our ideal sample size on an estimated pattern of means, standard deviations, and correlations between repeated measures. These estimates were matched as closely as possible to those in Study 1 in terms of ratio of means across cells, and their respective standard deviations. The sample size that we were able to recruit, however, was limited due to the requirement for in-person participation by community-dwelling participants of various ages. Therefore, we conducted a sensitivity analysis to determine the minimum effect size that our sample was powered to detect [30, 31]. We conducted the series of Monte Carlo simulations to test different magnitudes of possible mean differences and their estimated power to detect our hypothesized 3-way interaction. We stopped the procedure once we found a pattern of means that yielded a power estimate of approximately 0.80. The exact value for power was 0.83, the corresponding effects size (partial eta squared) was 0.06, and the latter indicated an estimate of minimum detectable effect size given our sample size. This suggests that if the true population effect size is 0.06 or greater, our sample size is sufficient to detect it; but if the true population effect size is smaller than 0.06, our sample would be underpowered (i.e., less than 0.83 and likely less than 0.80) to detect the effect.

#### Socio-demographics

We assessed socio-demographic characteristics and administered affective and cognitive measures (which are summarized in Table E in S2 File).

#### Eye tracking

Before participants viewed the faces and gathered information to make their decision, a trained research assistant connected the T60 eye tracker (Tobii Technology AB, Danderyd, Sweden) to measure gaze patterns toward the regions of interest on each of the three face images during the main tasks. The duration of visual fixation, on these regions, was our primary dependent measure. A fixation was defined as a gaze focused within 1° of the visual angle for at least 100 ms [32] inside a defined area of interest (AOI); namely, the square area surrounding each of the three emotional faces on the screen. As in Study 1, face images were 36 photographs of 36 middle-aged adults from the FACES database [25].

#### Choice of information source

As another dependent variable, we also calculated the average number of chosen happy, neutral, and angry faces by each participant for obtaining information to guide their decision about the allergy remedies.

#### Procedure

Participants were tested individually by trained research assistants in a quiet laboratory room using the same artificial light for each participant so as to guarantee that gaze tracking was not affected by the ambience. Participants first provided written informed consent, then completed demographic questions and a mood questionnaire. These questions were administered using the computerized software SoSci Survey [29]. Next, after a brief calibration of the gaze tracker, participants started the main task.

All participants began with a passive viewing condition in which they viewed a series of trials each displaying face images but did not gather any information or make any decisions. They were told they should view the faces naturally, as they would when looking through a magazine. Images were presented using E-Prime Version 2.0 [33] on a 17” monitor. Each photograph was 2.6” high and 2.1” wide. The distance between the photographs was 0.3”. Each trial first displayed a small fixation cross for one second to align gaze to the center of the screen. The passive viewing condition was first, and images remained on the screen for 10 seconds following the fixation cross. All 36 trials were displayed automatically.

**Fig 3:**
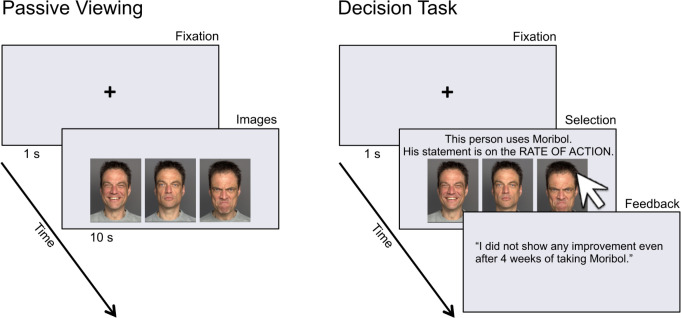
Sample trial; Study 2 (passive viewing = left, decision task = right).

Participants then completed the decision task condition (12 trials) in which they viewed faces similar to those from the passive condition but this time each of the three faces on the screen represented positive, neutral, and negative information about a particular allergy remedy (see Table 2 for examples). The majority of the faces shown had also been included in the passive viewing condition (i.e., the same individuals and each with the same three emotion expressions). For each trial, after a one-second fixation cross, the participants’ task was to obtain information by clicking on the face corresponding to the information they wanted to view. Their ultimate task was to continue gathering information that would allow them to make an optimal decision among the four allergy remedies, and additional text was shown on the screen as guidance (in contrast to the passive viewing condition where no text was displayed with the faces; see Fig 3 for an illustration of passive viewing and decision task conditions). See S3 File for instructions given to participants. After the 12 decision task trials, participants indicated their decision. Note that the decision was not the dependent variable of interest. Instead, the dependent variable was the percentage of happy vs. neutral vs. angry faces participants selected across these 12 choice trials. Lastly, after completing the post-test mood questionnaire, participants were thanked, debriefed, and reimbursed with 20 Swiss francs. The entire session lasted approximately 60–90 minutes. Procedures were consistent with the requirements of the local ethics committee (see Study 1).

#### Design

The experimental design was a 3 (age; younger, middle-aged, older) x 3 (valence; happy, neutral, angry) x 2 (condition; passive-viewing, decision task) mixed design (age as between-subjects factor; valence and condition as within-subjects factor). The dependent measures were eye tracking (fixation duration; detailed below) and valence of the face images chosen for receiving the corresponding information about the allergy medicines.

### Results

#### Fixation duration

Analyses were conducted after correcting for first view (i.e., the first fixation time was excluded from each trial in both conditions, as was time spent viewing instructions in the decision task). Whenever appropriate, Greenhouse-Geisser correction was applied to the degrees of freedom to account for deviations from sphericity. To test for differences in fixation duration by age, valence, and condition, a 3 x 3 x 2 repeated-measures ANOVA with valence (happy, neutral, angry) and condition (passive viewing, decision task) as within-subjects factors revealed a main effect for all three factors: valence, *F*(1.9, 315.68) = 252.18, *p* < .001, η_p_^2^ = .60; condition, *F*(1, 166) = 18.40, *p* < .001, η_p_^2^ = .10; and age, *F*(2, 166) = 8.84, *p* < .001, η_p_^2^ = .10. The specific main effect of valence favored longer fixation durations on neutral faces.

Main effects were qualified by a valence x age interaction, *F*(3.8, 315.68) = 11.81, *p* < .001, η_p_^2^ = .13; a condition x age interaction, *F*(2, 166) = 7.76, *p* < .01, η_p_^2^ = .09; and a condition x valence interaction, *F*(2, 332) = 93.35, *p* < .001, η_p_^2^ = .36. These lower-order interactions were qualified by a condition x valence x age interaction, *F*(4, 332) = 6.87, *p* < .001, η_p_^2^ = .08.

**Fig 4:**
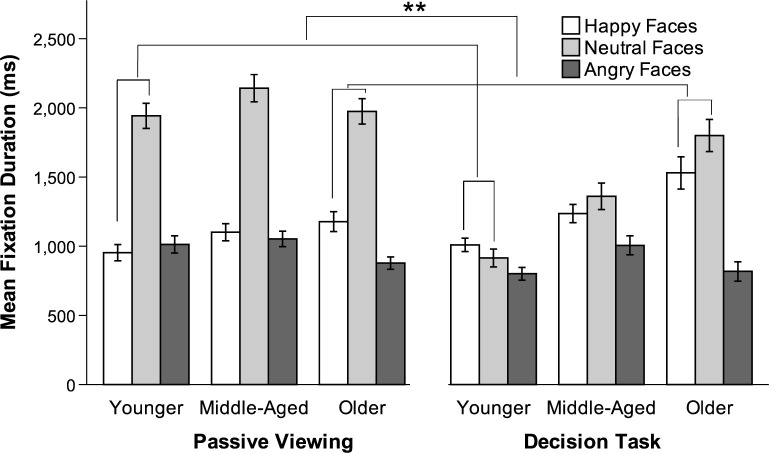
Mean fixation duration for passive viewing condition and decision task condition, Study 2. Younger = 18–30 years; Middle-Aged = 40–55 years; Older = 65–85 years. Error bars indicate SEM. ** *p* < .001.

To follow up on the significant three-way interaction, we examined age and valence in the passive viewing compared to the decision task condition to determine whether the effect of condition on gaze toward happy relative to angry faces differed for older versus younger adults. An interaction contrast revealed that the fixation duration difference between happy and angry faces from the passive viewing to the decision conditions was not significantly different for older (*M* = 413.204) than younger adults (*M* = 268.37). We then conducted two interaction contrasts to determine whether gaze toward neutral (relative to valenced) faces changed significantly from the passive viewing to the decision task conditions by age. The first contrast revealed that the fixation duration difference between happy and neutral faces from the passive viewing to the decision conditions was significantly smaller for older (*M* = 527.67) than for younger adults *M* = 1083.36, *t*(166) = 3.46, *p* < .001, *d* = 0.27 (see Fig 4). The second contrast revealed that the fixation duration difference between angry and neutral faces from the passive viewing to the decision conditions was significantly smaller for older (*M* = 114.47) than for younger adults *M* = 814.99, *t*(166) = 4.51, *p* < .001, *d* = 0.35 (see Fig 4). Older adults appear to maintain a preference toward neutral cues in the information condition whereas younger adults did not.

#### Image choice (happy, neutral, or angry)

**Fig 5:**
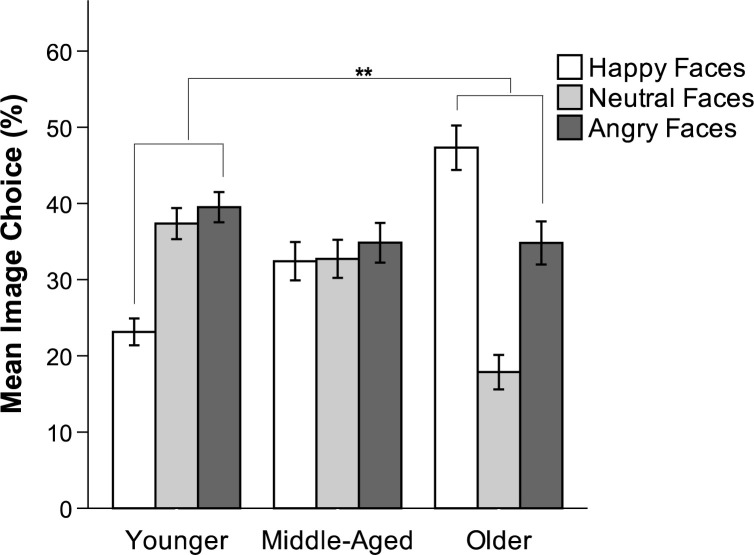
Mean image choice (percent happy, neutral, and angry), Study 2. Younger = 18–30 years; Middle-Aged = 40–55 years; Older = 65–85 years. Error bars indicate SEM. ** *p* < .001.

We tested for age and valence differences in the frequency of choosing a happy, neutral, or angry image to provide information about the decision options (i.e., percentage of trials where a happy versus an angry face was chosen, out of 12 total trials). Neutral faces were not included in the analysis as a third category, given that all participants’ number of choices across happy, neutral, and angry faces summed to 12 (i.e., their proportions summed to 1). Including all three categories in the analysis would make each category’s total necessarily dependent on the other two. A 3 x 2 repeated-measures ANOVA with valence (happy, angry) as the within-subjects factor revealed a main effect for age, *F*(2, 166) = 20.37, *p* < .001, η_p_^2^ = .20. Note, that this effect suggests that age groups differed with regard to neutral vs. valenced choices. There was no significant main effect for valence, *p* = .41, but a valence x age interaction, *F*(2, 166) = 11.04, *p* < .001, η_p_^2^ = .12. An interaction contrast revealed that the valence difference (proportion happy–proportion angry) was significantly larger for older (*M* = 0.13) than for younger adults (*M* = -0.16), *t*(166) = 4.70, *p* < .001, *d* = 0.36 (see Fig 5).

#### Brief discussion

Study 2 revealed that all age groups viewed a face with a neutral expression longest (the only exception being younger adults in the decision condition). When comparing the valenced information, all age groups looked longer at happy than at angry faces in both conditions (the only exception being younger adults in the passive viewing condition). Study 3 replicated the design from Study 2 but with different scenario; namely, a decision among vacation packages.

## Study 3

Study 3 used the same procedure and materials as Study 2 except that it involved information about a typically positive event, namely a decision between four vacation packages (instead of the more negative event of having to choose between allergy medicines in Study 2; here, the goal is to remove something negative—in this case allergies—compared to adding something positive—as in vacation packages). Our predictions regarding age, valence, and condition were the same as in Study 2. As in Study 2, we included both valenced and neutral faces as cues for decision-relevant information.

### Method

#### Participants

Participants (*N* = 181) were recruited from the larger Boston area (USA) using a participant database, internet advertisements, newspaper and monthly consumer ad booklets, and an Introductory Psychology Subject Pool. We aimed at 60 participants per age group. However, we had to exclude *n* = 36 participants (20%) whose eye movements were not trackable in at least 25% of the trials. This resulted in a final sample size of *N* = 145. The sample contained *n* = 52 younger adults (18–30 years, *M* = 20.22, *SD* = 1.92, 84.1% female), *n* = 45 middle aged adults (40–59 years, *M* = 51.29, *SD* = 5.59, 42.2% female), and *n* = 48 older adults (60–91 years, *M* = 70.94, *SD* = 7.84, 76.6% female).

As in Study 2, the sample size was limited due to the requirement for in-person participation by community-dwelling participants of various ages. Therefore, we again conducted a sensitivity analysis to determine the minimum effect size that our sample was powered to detect [30, 31]. Using the same pattern of expected means as Study 2, we conducted the series of Monte Carlo simulations to test different magnitudes of possible mean differences and their estimated power to detect our hypothesized 3-way interaction. We stopped the procedure once we found a pattern of means that yielded a power estimate of approximately 0.82. The corresponding effects size (partial eta squared) was 0.06, indicating an estimate of minimum detectable effect size given our sample size. Similar to Study 2, this sample size can provide meaningful results.

All participants provided written informed consent, and all procedures were approved by the Brandeis University IRB (protocol number 09036).

#### Measures

Prior to the eye tracking session and after providing informed consent, participants filled out a series of demographic, affective, and personality questionnaires. Demographic information included age, ethnicity, religious affiliation, education level, and self-rated health. Upon completing these questions, participants performed three visual exams: the Snellen 20/20 E-Chart for far vision, the Pelli-Robson Chart for contrast sensitivity [34], and the Rosenbaum Chart for near vision [35]. These measures are summarized in Table F in S2 File.

#### Procedure and experimental instructions

Participants were tested individually by trained research assistants. They sat in front of a 17” computer monitor in order to begin the eye tracking tasks. The procedure for eye-tracking set-up was identical to Study 2. Gaze duration was measured by trial, for each of the three emotion valences, by calculating fixation duration on the respective face as a percentage of total fixation duration during that trial (including fixations outside of the three facial stimuli). A fixation was defined as a gaze focused for at least 100 ms within 1° of the visual angle [32].

Participants performed the same two eye-tracking conditions as in Study 2 (passive viewing first, followed by the decision task). Specifically, in the decision task, participants were instructed that they were, again, going to view images of people who have just returned from their vacation to specific Caribbean islands. However, this time they were asked to choose one of the faces. The choice permitted them to get more information about the vacation experience of that person.

Participants then performed a series of cognitive and perceptual tasks as well as personality questionnaires after the eye tracking session that are not part of this article (See Table F in S2 File). Participants were then debriefed as to the nature of the study and given their allotted compensation ($10 or 1 course credit for students; $15 for adults recruited from the community). The experimental session lasted between 60–90 minutes.

### Results

#### Fixation duration

Whenever appropriate, Greenhouse-Geisser correction was applied to the degrees of freedom to account for deviations from sphericity. To test for differences in fixation duration by age, valence, and condition, a 3 x 3 x 2 repeated-measures ANOVA with valence (happy, neutral, angry) and condition (passive viewing, decision task) as within-subjects factors revealed a main effect for valence, *F*(1.72, 244.3) = 85.06, *p* < .001, η_p_^2^ = .38; and condition, *F*(1, 142) = 204.37, *p* < .001, η_p_^2^ = .63. Main effects were qualified by a valence x age interaction, *F*(3.44, 244.3) = 3.0, *p* = .025, η_p_^2^ = .04; and a condition x valence interaction, *F*(1.87, 266.02) = 120.44, *p* < .001, η_p_^2^ = .46.

**Fig 6:**
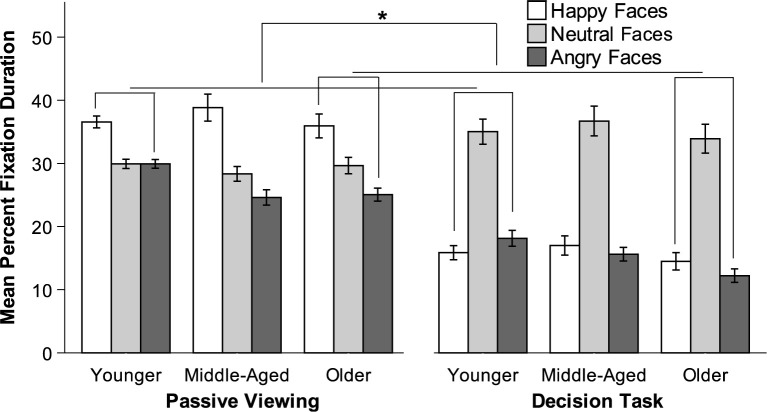
Mean fixation duration for passive viewing condition and decision task condition, Study 3. Younger = 18–30 years; Middle-Aged = 40–59 years; Older = 60–91 years. Error bars indicate SEM. * *p* < .05.

We followed up on the significant valence x age interaction given that it was of interest in testing our predicted effects. We examined whether differences in gaze duration toward happy versus angry faces differed between older and younger adults, regardless of condition (i.e., passive viewing vs. decision task). An interaction contrast revealed that fixation duration difference toward happy over angry faces was greater for older adults (*M* = 6.67) than for younger adults (*M* = 2.31), *t*(142) = 2.10, *p* = .038, *d* = 0.17 (see Fig 6). We then conducted two interaction contrasts to determine whether gaze toward neutral (relative to valenced) faces differed by age. The first contrast revealed that fixation duration difference toward neutral over happy faces did not differ between older adults (*M* = 7.48) and younger adults (*M* = 6.14), *t*(142) = 0.52, *p* = .60, *d* = 0.04. The second contrast revealed that fixation duration difference toward neutral over angry faces was greater for older adults (*M* = 14.14) than for younger adults (*M* = 8.45), *t*(142) = 3.16, *p* = .002, *d* = 0.26 (See Fig 6). Older adults appear to prefer neutral cues over angry cues, across both conditions, more so than younger adults.

#### Image choice (happy, neutral, angry)

**Fig 7:**
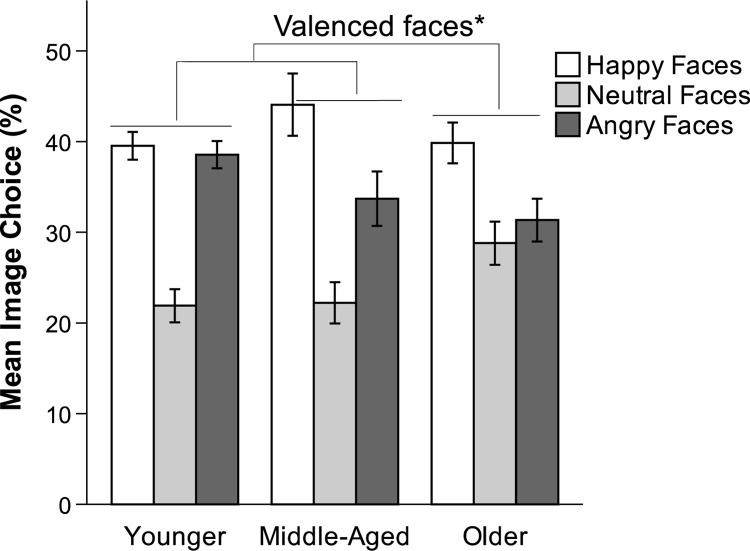
Mean image choice (percent happy, neutral, and angry), Study 3. Younger = 18–30 years; Middle-Aged = 40–59 years; Older = 60–91 years. Error bars indicate SEM. * *p* < .05.

We tested for age and valence differences in the frequency of choosing a certain facial expression (i.e., percentage of trials where a happy versus an angry face was chosen out of 12 total trials). As in Study 2, neutral faces were not included in the analysis as a third category, given that all participants’ number of choices across happy, neutral, and angry faces summed to 12 (i.e., their proportions summed to 1). Two additional participants were excluded from these analyses due to missing data for one of the 12 choice trials. A 3 x 2 repeated-measures ANOVA with valence (happy, angry) as the within-subjects factor revealed a main effect for valence, *F*(1, 140) = 6.76, *p* = .01, η_p_^2^ = .05, and for age, *F*(2, 140) = 3.31, *p* = .039, η_p_^2^ = .05. The valence x age-group interaction was not significant (*p* = .20), indicating no age difference in the proportion of happy compared to angry faces selected. However, the significant main effect of age indicates that age groups differed in the proportion of valenced faces selected (i.e., happy and angry) compared to neutral faces. Thus, regardless of valence (happy versus angry), there was a significant difference among the three age categories regarding the number of valenced faces chosen. As a hypothetical example, if out of the total of 12 trials older adults chose ten valenced faces on average, while younger adults chose on average only two valenced faces out of the total of 12 trials, this would imply that older adults had chosen two neutral faces on average (12 total– 10 valenced = 2 neutral) compared to younger adults’ 10 neutral faces on average (12 total– 2 valenced = 10 neutral). Thus the analysis still accounts for neutral choices although they were not technically included in the ANOVA. An interaction contrast revealed no significant differences between percent happy and percent angry faces chosen by older (0.086) versus younger adults (0.003, n.s.). We therefore conducted a second interaction contrast to determine specific age differences, comparing younger and middle-aged adults to older adults. The contrast revealed that younger and middle-aged adults chose a greater proportion of valenced faces (*M* = 0.39; *SD* = 0.16) than did older adults (*M* = 0.36; *SD* = 0.16), *t*(140) = 2.56, *p* = .011, *d* = 0.22 (see Fig 7). Said differently, older adults chose a significantly greater proportion of neutral faces than did younger and middle-aged adults.

### Brief discussion

Consistent with Study 2, findings from Study 3 indicate that, regardless of age, adults gazed most at neutral faces in the decision task condition. While we cannot rule out that this might be due to the experimental set-up (neutral face was displayed in the middle of the screen for both Studies 2 and 3), a similar preference toward neutral images was found in Study 1, which involved only self-report and not gaze. Thus, it is unlikely that image position confounded any effects of valence. In addition to the gaze findings, older adults chose negative information less frequently than younger or middle-aged adults when deciding about a vacation package. Finally, it is possible that older adults were less negative-avoidant because they were gathering information on a positive event (i.e., a vacation). However, similar findings for gathering information on allergy remedies (Study 2) suggest that this positivity pattern is not restricted to positive events.

## General discussion

Whom would you consult if you had to decide among different vacation packages or allergy medications–a happy, angry, or neutral person? In contrast to our hypotheses of age-differential focus on positive vs. negative source information, the current studies suggest that people do not necessarily prefer to obtain information from an emotional source (happy or angry). Instead, people seem to prefer neutral information sources and avoid negative ones.

Regarding age-related differences in information preference, our findings were contrary to predictions. We had hypothesized that older adults prefer happy faces over angry faces for passive viewing conditions but the reverse pattern for decision task conditions, i.e., that older adults prefer information provided by angry faces over information provided by happy faces in order to avoid negative outcomes. We expected younger adults to show little or no preference for happy faces across both passive viewing and decision task conditions. Our current findings revealed a positive preference in older adults across both conditions. Gaze patterns from Studies 2 and 3 suggest that older adults prefer positive over angry faces, including in the decision task conditions. Thus, age-related shifts in goal orientation toward an increasing importance of preventing losses or adverse outcomes [15–19] did not appear to manifest in the decision tasks.

### Importance of neutral information

Our findings demonstrate clear reasons for why it is important to include “neutral” as an option when studying preferences for valenced information. First, when faces served as cues for decision-related information, people of all ages showed a stronger-than-expected preference for neutral information. This was evident in gaze patterns across Studies 2 and 3, and to some extent in self-reports from Study 1 (health domain). Older adults maintained a stronger preference for neutral information sources relative to angry (and in some cases happy) as compared to younger adults—particularly in decision contexts. This suggests that, to fully understand age differences in preferred valence of information sources, neutral sources should be studied along with positive and negative sources. The current studies suggest that people may value non-valenced sources, and that the preference for neutral sources increases with advancing age.

In addition, including neutral as an option in studies on information preference (especially with regard to age differences) clarifies the extent to which anger-avoidance may motivate people to prefer positive information sources. That is, although people seem to prefer “happy” sources if they are only given an “angry” source as the alternative, they may actually try to *avoid negative* sources. These preferences seem to be further be moderated by age. Older adults in Study 1 reported a stronger angry versus happy difference than younger adults, but this age pattern was reversed when text was used as the information source. In this case, younger adults reported a stronger negative versus positive difference than older adults. Thus, because we included a neutral option in all cases, we were able to detect a stronger avoidance of angry faces as informational sources in older adults. While we did not find this effect for the choice data in Studies 2 and 3, we did see this effect in older adults across the gaze data and the evaluation data from Study 1. Older adults avoided angry stimuli, thereby favoring either neutral *or* happy stimuli.

Had we excluded neutral sources, our results would have likely suggested a preference in older adults *toward* happy information sources rather than a preference *away* from angry information sources. In the same vein [36], have argued for the importance of incorporating neutral stimuli in studying emotional preference. Similarly [37], have pointed to the importance of measuring valence preference (positive or negative) relative to neutral stimuli so that independent comparisons for each effect are possible as well as separate consideration of their magnitudes. Importantly, the inclusion of neutral stimuli demonstrated across studies that older adults showed a strong preference for neutral faces. This might reflect a tendency to trust such sources more as the information a neutral source provides is less clouded by the emotional state. However, in line with Charles’ SAVI model [38], this pattern of results could also be driven by older adults’ stronger avoidance of angry faces due to their high arousal [39] that is difficult to downregulate for older adults. It is also possible that, although the FACES database was validated as to the ratings of the faces as emotional (valenced) or neutral [15], neutral faces may still have been *perceived* as somewhat *emotional* [22]. Thus, we cannot rule out that longer gaze times toward the neutral faces could in part reflect an attempt to discern their facial expression. However, this possibility seems unlikely in our studies—particularly the decision tasks in Studies 2 and 3 where participants were told specifically that they would choose whether they wanted a piece of information that” has made the person angry, has made them happy, or has made them neutral.” Thus they were told what emotion each image conveyed, and their task was only to decide which piece of information they wanted.

Some evidence suggests that older adults tend to avoid angry stimuli compared to *other types of negative stimuli*. For instance, in a gaze study older adults showed a preference toward happy faces and away from angry faces—but again no significant preference regarding sad or fearful faces [12]. Taken together, older adults may avoid stimuli that symbolize anger—such as angry faces as in our current studies—but may not avoid stimuli expressing sadness or fear. Future studies are needed to compare different specific emotions in order to investigate potential differences between anger and other negative emotion expressions. Thus our current findings, revealing what appear to be positivity effects with age, may be at least partly due to our use of angry faces as the source of negative information in the decision tasks. Further research is needed to clarify the specific factors in decision contests that lead to preference for positive versus negative information in older adults.

### Potential explanations for avoidance of negative information in decision tasks

Results did not support our expectation that older adults focus on negative information to avoid losses. One possible explanation for these findings is that older adults did not view the decisions in the hypothetical scenarios concerning allergy remedies and vacation packages as opportunities to prevent losses. Moreover, selecting a computerized face as a source of information might have distracted participants from the potential adverse outcomes of the scenarios. They might not have felt any “real” threat of losses or adverse outcomes in these tasks, and thus not have been motivated to seek information that could prevent adverse outcomes. Note, that the scenarios suggested that the anger expressed in our stimuli was not directed at the participant but signaled negative information about the decision options.

A possible explanation for the current finding is that older adults do not value angry faces as a useful source of information. In fact, in Study 1, older adults’ preference for positive over negative information sources was most pronounced when faces served as the information source. Moreover, this preference for positive over angry faces was generally larger for older adults than for younger adults. In contrast, preference for positive over negative *text* sources was *smaller* for older adults than for younger adults. Thus, faces, as an information source, might be less likely to elicit negativity effects with age in a decision-making context. It might be that older adults doubt particularly that an angry source is able to convey information that will help them in their decision. This would also be consistent with the choice data in Studies 2 and 3, which again showed stronger preference for happy over angry information sources in older adults than in younger adults.

While older adults seem to avoid angry faces as information sources, Study 1 suggests no age effect in avoiding negative text-based information sources. Consistent with this, prior studies suggest that older adults may even value negative text-based sources in certain decision contexts. Compared to younger adults, older adults showed a stronger preferential recall for negative text-based information over positive text-based information in decision tasks [5], and showed no preferential attention for positive over negative text-based information in a personally-important decision task [20]. Our current findings, revealing only positivity effects with age, may be at least partly due to our use of angry faces as the source of negative information in the decision tasks (though it may be hard to find information associated with other negative emotions like sadness). With this, our findings add to the extant evidence that certain decision contexts elicit no age-related positivity effects or even age-related negativity effects, while other contexts elicit age-related positivity effects. Again, further research is needed to clarify the specific factors leading to preference for positive versus negative information in older adults when making important decisions.

### Limitations and conclusions

The current studies have several noteworthy limitations. First, the fixed order in Studies 2 and 3 (with passive viewing first) renders it impossible to rule out order effects. We believe this limitation is difficult to avoid, however, because counterbalancing the order would have compromised the “passive” nature of the passive viewing condition. Specifically, it would no longer be passive to those viewing the faces a second time who have already made a decision based on them in the previous round. Also, a between-subjects design—in which participants are randomly assigned to either the passive viewing or the decision task condition but not both—would be necessary to prevent order effects. Using such a design in the current study would have required nearly twice the sample size for Studies 2 and 3, but is an important next step for future research. Replicating our findings using a between-subjects design would be necessary to rule out potential order effects on our results.

Another limitation is that faces were always presented in the same arrangement on the screen: happy, neutral, and angry (left, middle, and right, respectively). We chose this fixed order so as to minimize the necessity to orient oneself when seeing the visual display. However, this order may have contributed to the tendency to gaze at neutral faces (always the middle of the three). Note, however, that the same order was also used in Study 1 which did not measure gaze, but neutral preferences were still found in ratings of overall informational value in the health domain. Still, it is possible that position on the screen affected self-report as well as eye gaze, so future studies should rule out this possibility by randomizing the order of the information sources. However, note that the first fixation in each trial was excluded from the analysis in Study 2. Moreover, there was a conceptual reason for not randomizing the order on the screen: we were not interested in where people glance when searching for a cue, but rather where they look when given three options. Thus, our paradigm does not tap into *how* people search, nor *how long* does it take them, but rather *where* they look in a known array of cues. Still, future research should examine whether randomizing position on the screen yields similar results.

A third limitation is that Studies 1 and 2 were conducted in Switzerland, whereas Study 3 was conducted in the United States. Please note, however, that typical studies that are conducted within one national context simply assume that the results are generalizable across cultural contexts. In our view, demonstrating the same pattern of results between Studies 2 and 3 strengthens rather than weakens the robustness of the findings.

Future research is also needed to investigate *why* people might value information coming from someone in a neutral state as opposed to a happy or angry state. Findings from Study 1 suggest that people perceive the most informational value in sources with a neutral facial expression but do not perceive neutral sources as more helpful or trustworthy than emotionally valenced sources. This leaves open the question about the underlying reasons for the preference of neutral information. It may be that people do not have access to their reason for a preference when asked to provide it and self-reported interest in information does not always correlate with actual behavior [23]. It would also be consistent with our gaze findings—which may constitute an implicit measure of information preference compared to self-reports which capture explicit preferences. These methods might tap into different aspects of preferences for decision-relevant information, or they may represent different ways to measure preference that may better capture the construct when used together.

A final potential insight contributed by our findings of a preference for neutral information sources is that they may reflect participants’ physiological state as they completed the decision tasks. While arousal levels were not assessed during the tasks, multiple studies have linked higher arousal during encoding to better attention and recall for valenced than for neutral stimuli [40–43]. Further research could investigate whether lower arousal levels during decision tasks may be linked to greater preference for neutral information sources.

Taken together, the current studies show that people show a greater-than-expected preference to get information from neutral sources as compared to positive or negative sources when they are asked to make a decision. It may be that the most unbiased information comes from those in a neutral state. Our findings suggest that the value of neutral states has been underestimated in previous research on aging and emotion.

## Supporting information

S1 File Supporting information about Study 1 results. Reports results when helpfulness, trustworthiness, and informational value were analyzed separately.
(PDF)

S2 File Supporting tables. Contains Tables A-F with additional descriptive statistics and participant characteristics that were not presented in the main article.
(PDF)

S3 File Instructions to participants. Study 2 passive viewing and decision tasks.
(PDF)

S1 Appendix Six sample screens for health scenario; six sample screens for vacation scenario. (PDF)
