# Supplementary material for: Age and information preference: Neutral information sources in decision contexts
Source: PLoS One. 2022 Jul 18;17(7):e0268713. doi: 10.1371/journal.pone.0268713 (PMC9292105; doi:10.1371/journal.pone.0268713)
Supplement: S1 File — Reports results when helpfulness, trustworthiness, and informational value were analyzed separately. (PDF) [file pone.0268713.s001.pdf]

# S1 File: Supporting information about Study 1 results

**Study 1: Helpfulness, trustworthiness, and informational value analyzed separately**

***Helpfulness.*** Age differences in how helpful the participants rated the information for the three information valences in both conditions (i.e., faces and text) and in both domains (health and vacation) were examined in a 3 (age: younger, middle-aged, older) x 3 (valence: happy, neutral, angry) x 2 (condition: faces, text) x 2 (domain: health, vacation) repeated measures ANOVA with age group as the between subject-factor and the other three as within-subjects factors. Whenever appropriate, Greenhouse-Geisser correction was applied to the degrees of freedom to account for deviations from sphericity.

Results revealed a main effect of valence, $F(1.62, 290.31) = 540.61, p < .001, \eta_p^2 = .75$. The main effect was qualified by a valence x domain interaction, $F(1.90, 340.34) = 41.42, p < .001, \eta_p^2 = .19$, and a condition x domain interaction, $F(1, 179) = 4.90, p < .05, \eta_p^2 = .03$. These lower-order interactions were qualified by an age x valence x condition interaction, $F(2.91, 260.05) = 5.31, p < .01, \eta_p^2 = .06$. We investigated the three-way interaction further by testing for age differences by valence within each of the two domains. For the health domain, all age groups rated angry faces as less helpful than other faces. However, older adults did so to a greater degree (mean difference = 2.54) than younger and middle-aged adults (mean difference = 1.83), $t(179) = 2.69, p = .008, d = 0.20$. Participants also rated negative text as less helpful than positive or neutral text, but older adults did so to a lesser degree (mean difference = 1.68) than younger and middle-aged adults (mean difference = 2.53), $t(179) = 2.97, p = .003, d = 0.22$.

There were no significant age differences for the vacation-related ratings of text pieces, but a significant condition x valence interaction emerged, $F(1.49, 266.61) = 4.51, p = .02, \eta_p^2 =$

.03. Simple main effects tests of condition within valence levels showed that neutral faces were rated as less helpful ($M = 4.59$) than neutral text ($M = 4.86$), $t(179) = 2.84$, $p = .005$, $d = 0.21$.

***Trustworthiness***. Age differences trustworthiness ratings of the information were examined using the same ANOVA procedure as for helpfulness. Results revealed main effects for valence, $F(1.59, 284.30) = 284.20$, $p < .001$, $\eta_p^2 = .61$; condition, $F(1, 179) = 17.83$, $p < .001$, $\eta_p^2 = .09$; and domain, $F(1, 179) = 6.60$, $p < .05$, $\eta_p^2 = .04$.

These main effects were qualified by a valence x condition interaction, $F(1.75, 313.44) = 25.23$, $p < .001$, $\eta_p^2 = .12$; a domain x age interaction, $F(2, 179) = 3.99$, $p < .05$, $\eta_p^2 = .04$; a domain x valence interaction, $F(2, 358) = 32.67$, $p < .001$, $\eta_p^2 = .15$; and a domain x condition interaction, $F(1, 179) = 11.00$, $p = .001$, $\eta_p^2 = .06$. These interactions were further qualified by a three-way interaction for age x valence x condition, $F(3.51, 313.94) = 4.60$, $p < .01$, $\eta_p^2 = .05$. To further investigate the three-way interaction, we tested for age differences by valence within each of the two domains. For the health domain, all age groups rated negative sources as less trustworthy than non-negative sources, but for the text condition, older adults did so to a lesser degree (mean difference = 0.64) than did younger and middle-aged (mean difference = 1.36) adults, $t(179) = 2.94$, $p = .004$, $d = 0.22$. For the vacation domain, again all age groups rated negative sources as less trustworthy than neutral or positive sources, but for the faces condition, older adults did so to a greater degree (mean difference = 2.07) than younger and middle-aged adults (mean difference = 1.06), $t(179) = 2.27$, $p = .025$, $d = 0.17$.

***Informational value***. Age differences regarding how much they would like to ask this person for information (or obtain this text) to make a decision, were examined again using the same approach as the previous two questions. Results revealed a main effect of valence, $F$(1.31,

234.36) = 443.60, $p < .001$, $\eta_p^2 = .71$; condition, $F(1, 179) = 10.71$, $p = .001$, $\eta_p^2 = .06$; and domain, $F(1, 179) = 4.75$, $p < .05$, $\eta_p^2 = .03$.

Main effects were qualified by a valence x condition interaction, $F(1.37, 245.97) = 20.87$, $p < .001$, $\eta_p^2 = .05$; and a valence x domain interaction, $F(1.78, 318.65) = 30.00$, $p < .001$, $\eta_p^2 = .14$. These interactions were further qualified by a three-way interaction for age x valence x condition, $F(2.82, 251.98) = 3.58$, $p < .05$, $\eta_p^2 = .04$. To investigate the three-way interaction, as with our analyses for helpfulness and trustworthiness, we conducted interaction contrasts to test for age differences by valence. Within the health domain, all age groups rated negative sources as less valuable than non-negative sources, but for the text condition, older adults did so to a lesser degree (mean difference = 1.36) than did younger and middle-aged adults (mean difference = 2.13), $t(179) = 2.81$, $p = .006$, $d = 0.21$.

This pattern in ratings suggests a strong preference to obtain information from people who are in a neutral emotional state. However, this neutral preference did not seem to extend to the texts. Given the higher ratings of informational value for neutral than for happy faces, we conducted a follow-up interaction contrast to test whether this preference for a neutral source was significantly larger for faces than for text. Within the health domain, ratings of informational value for neutral faces were higher than the averaged ratings for angry and happy faces (mean difference = 1.42), and this difference was significantly larger than the same difference for the text condition (mean difference = 0.78), $t(179) = 5.82$, $p < .001$, $d = 0.43$.

Within the vacation domain, a similar follow-up interaction contrast revealed that ratings of informational value for neutral faces were higher than the averaged ratings for angry and happy faces (mean difference = 0.90), and again this difference was significantly larger than the same difference for the text condition (mean difference = 0.47), $t(179) = 3.47$, $p = .001$, $d = 0.26$.
