# Supplementary material for: Age and information preference: Neutral information sources in decision contexts
Source: PLoS One. 2022 Jul 18;17(7):e0268713. doi: 10.1371/journal.pone.0268713 (PMC9292105; doi:10.1371/journal.pone.0268713)
Supplement: S2 File — Contains Tables A-F with additional descriptive statistics and participant characteristics that were not presented in the main article. (PDF) [file pone.0268713.s002.pdf]

S2 File: Supporting tables

Table A

Study 1 Information Value Score: Health Domain

Measure	Younger		Middle-Aged		Older	
	M	*SD*	*M*	*SD*	*M*	*SD*
Faces						
Happy	4.89	1.00	4.75	1.12	4.86	0.99
Neutral	4.71	1.10	5.00	1.13	4.98	1.17
Angry	2.98	1.42	3.13	1.49	2.70	1.50
Text						
Happy	5.27	1.12	5.10	1.11	5.16	1.22
Neutral	5.04	1.15	4.93	1.18	4.85	1.22
Angry	3.03	1.06	3.12	1.18	3.78	1.33

Table B

Study 1 Information Value Score: Vacation Domain

Measure	Younger		Middle-Aged		Older	
	M	*SD*	*M*	*SD*	*M*	*SD*
Faces						
Happy	5.18	0.89	5.11	0.97	5.15	0.75
Neutral	4.45	1.09	4.48	1.18	4.62	1.12
Angry	3.11	1.48	3.09	1.59	2.77	1.39
Text						
Happy	5.49	0.98	5.19	0.95	5.24	1.17
Neutral	4.75	1.11	4.57	1.02	4.36	1.31
Angry	3.12	1.12	3.22	1.22	3.10	1.24

Table C

Study 2 Gaze Duration and Image Choice

	Younger		Middle-Aged		Older	
Measure	*M*	*SD*	*M*	*SD*	*M*	*SD*
Gaze Duration (Passive)						
Happy	953.19	447.46	1100.91	462.42	1177.55	535.50
Neutral	1942.41	696.23	2142.98	735.37	1974.82	682.22
Angry	1012.86	479.25	1052.27	412.03	878.03	334.98
Gaze Duration (Decision)						
Happy	1009.51	368.95	1235.41	489.12	1530.27	871.93
Neutral	915.37	494.06	1361.10	710.13	1799.86	869.67
Angry	800.81	350.02	1006.37	507.73	817.54	526.59
	ms		ms		ms	
Image Choice						
Happy	23.13	13.43	32.42	18.68	47.32	21.85
Neutral	39.51	15.10	34.85	19.32	34.82	21.21
Angry	37.36	15.47	32.73	18.56	17.86	16.85
	%		%		%	

Table D

Study 3 Gaze Duration and Image Choice

Measure	Younger		Middle-Aged		Older	
	M	*SD*	*M*	*SD*	*M*	*SD*
Gaze (Passive and Decision)						
Happy	26.52	7.62	28.71	12.32	26.13	11.85
Neutral	32.67	9.95	32.87	11.23	33.60	12.24
Angry	24.22	7.57	20.31	7.68	19.46	7.63
Image Choice						
Happy	39.26	10.99	44.07	23.07	39.86	15.31
Angry	38.94	11.04	33.70	20.18	31.34	16.02
Valenced	39.10	11.01	38.89	21.62	35.60	15.67
	%		%		%	

For Study 2, affective questionnaires included assessments of current mood (PANAS; Watson, Clark, & Tellegen, 1988), trait optimism (LOT; Scheier & Carver, 1985), state and trait anxiety (STAI Y-1 and Y-2; Speilberger, Gorsuch, Lushene, Vagg, & Jacobs, 1983), and future time perspective (FTP Scale; Carstensen & Lang, 1996). Cognitive assessments included tests of fluid intelligence (digit span from the HAWIE; Demuth, 1983; and digit symbol substitution from the WAIS-R; Weschler, 1981). These constructs are not considered in this article but are summarized in Table E.

Table E

Participant Characteristics Study 2

Measure	Younger		Middle-Aged		Older	
	M	*SD*	*M*	*SD*	*M*	*SD*
LOT	16.14	3.26	15.33	3.96	17.72	2.54
FTP	55.12	7.27	41.98	11.26	36.43	12.94
PANAS Positive Affect	46.88	6.75	49.65	7.52	55.06	6.69
PANAS Negative Affect	16.72	6.00	16.91	7.25	13.06	3.74
STAI-S	72.90	7.62	71.71	8.43	76.44	7.19
STA-T	74.55	10.67	73.65	10.95	67.67	8.67
Digit Span	18.43	4.04	16.87	4.47	16.07	3.44
Digit Symbol	63.03	10.57	52.15	11.31	43.87	8.06

For Study 3, affective questionnaires included assessments of current mood (PANAS; Watson, Clark, & Tellegen, 1988), trait optimism (LOT; Scheier & Carver, 1985), state and trait anxiety (STAI Y-1 and Y-2; Speilberger, Gorsuch, Lushene, Vagg, & Jacobs, 1983), depressive symptoms (CES-D; Radloff, 1977), and future time perspective (FTP Scale; Carstensen & Lang, 1996). In addition, participants performed a series of cognitive and perceptual tasks as well as personality questionnaires after the eye tracking session. These measures screened for symptoms of dementia (MMSE; Folstein, Folstein, & McHugh, 1975), and included tests of fluid intelligence (forward digit span, backward digit span, and digit symbol substitution from the WAIS-R; Weschler, 1981), and crystallized intelligence (Shipley Vocabulary; Zachary, 1986). Finally, participants filled out a final series of questionnaires assessing affiliation tendencies and

sensitivity (Merhabian Scale; Mehrabian, 1970), need for cognition (NCS; Cacioppo, Petty, & Kao, 1984), and life satisfaction (SWLS; Diener, Emmons, Larsen, & Griffin, 1985). These constructs are not considered in this article but are summarized in Table F.

Table F

Participant Characteristics Study 3

Measure	Younger		Middle-Aged		Older	
	M	*SD*	*M*	*SD*	*M*	*SD*
Mehrabian AT	118.21	18.55	117.78	12.12	118.38	11.72
Mehrabian SR	107.69	21.73	108.69	13.04	108.75	12.38
STAI-S	35.88	9.54	35.27	10.86	30.74	8.06
STAI-T	39.06	9.79	36.49	10.45	34.88	8.59
PANAS Positive Affect	29.90	6.97	32.42	8.95	32.56	7.25
PANAS Negative Affect	15.90	6.01	13.93	4.31	13.23	4.36
Optimism	11.56	2.70	10.07	3.29	9.96	2.14
Pessimism	11.15	2.78	11.18	3.97	12.06	3.45
LOT	22.71	4.30	21.24	6.64	22.02	4.37
CES-D	12.33	7.45	10.38	8.84	9.15	6.50
FTP	55.50	7.96	46.33	12.54	36.79	12.14
NCS	66.35	10.20	63.40	12.13	64.27	11.49
SWLS	26.39	5.73	20.69	7.59	23.44	6.08
MMSE	29.21	0.85	29.69	0.63	29.06	1.09
Forward Digit Span	7.29	1.49	7.09	1.18	7.06	1.34
Backward Digit Span	5.73	1.40	5.27	1.21	5.54	1.25
Shipley Vocabulary	14.69	2.25	15.18	2.09	16.81	2.18
Digit Symbol	72.60	8.78	57.36	11.21	53.65	10.55
Self-rated Health	3.98	0.75	3.76	0.93	3.75	1.00
Far visual acuity	22.19	6.06	35.89	17.97	35.83	15.28
Near visual acuity	21.54	2.33	42.25	31.91	49.17	60.11
Contrast sensitivity	1.62	0.10	1.63	0.08	1.53	0.16

References

Cacioppo JT, Petty RE, Kao CF. The Efficient Assessment of Need for Cognition. *Journal of Personality Assessment*. [Online] 1984; 48(3): 306–307. doi:10.1207/s15327752jpa4803_13

Carstensen LL, Lang FR. Future Time Perspective Scale. Stanford, CA: Stanford University; 1996.

Demuth W. Entwicklung einer HAWIE-Kurzform für Untersuchung und Begutachtung [Development of a HAWIE (Hamburg Wechsler Intelligence Test for Adults) short form for examination and assessment]. Schweiz Arch Neurol Neurochir Psychiatr. 1983; 132(1): 89-100. German. PMID: 6857167.

Diener ED, Emmons RA, Larsen RJ, Griffin S. The satisfaction with life scale. Journal of Personality Assessment. 1985; 49(1): 71-75.

Folstein MF, Folstein SE, McHugh PR. "Mini-mental state": a practical method for grading the cognitive state of patients for the clinician. Journal of Psychiatric Research. 1975; 12(3): 189-198.

Mehrabian A. The development and validation of measures of affiliative tendency and sensitivity to rejection. *Educational and Psychological Measurement*. 1970; 30(2): 417–428. doi: 10.1177/001316447003000226

Radloff LS. The CES-D scale: A self-report depression scale for research in the general population. Applied Psychological Measurement. 1977; 1(3): 385-401.

Scheier MF, Carver CS. Optimism, coping, and health: assessment and implications of generalized outcome expectancies. Health Psychology. 1985; 4(3): 219-247.

Spielberger CD, Gorsuch RL, Lushene RE, Vagg PR, Jacobs GA. Manual for State-Trait

Anxiety Inventory. Palo Alto, CA: Consulting Psychologists; 1983.

Watson D, Clark LA, Tellegen A. Development and validation of brief measures of positive and negative affect: The PANAS scales. Journal of Personality and Social Development. 1988; 54: 1063-1070.

Wechsler D. Manual for the Wechsler Adult Intelligence Scale-Revised. New York, NY: Psychological Corporation; 1981.

Zachary R. Shipley Institute of Living Scale, revised manual. Los Angeles, CA: Western Psychological Services; 1986.
