# Supplementary material for: Age and information preference: Neutral information sources in decision contexts
Source: PLoS One. 2022 Jul 18;17(7):e0268713. doi: 10.1371/journal.pone.0268713 (PMC9292105; doi:10.1371/journal.pone.0268713)
Supplement: S3 File — Study 2 passive viewing and decision tasks. (PDF) [file pone.0268713.s003.pdf]

S3 File: Instructions to participants

(Study 2 passive viewing and decision tasks)

For this part of the study, you will be looking at pictures of people who have taken different allergy medicines.

Passive viewing task:

You will see a series of faces.

Please look at the groups of faces naturally, as you would when looking through a magazine.

Decision task:

Below is an example of a similar task.

This person used the allergy remedy ZARTEG. They can tell you something about the PRICE.

You can choose whether you want a piece of information that has made the person angry, has made them happy, or has made them neutral.

Click one of the three images of the person to select the appropriate information.

[Sample Trial]:

This person used the allergy remedy MORIBOL.

They can tell you something about the AVAILABILITY.
