# Supplementary material for: Age and information preference: Neutral information sources in decision contexts
Source: PLoS One. 2022 Jul 18;17(7):e0268713. doi: 10.1371/journal.pone.0268713 (PMC9292105; doi:10.1371/journal.pone.0268713)
Supplement: S1 Appendix — (PDF) [file pone.0268713.s004.pdf]

S1 Appendix: Six sample screens for health scenario; six sample screens for vacation scenario.

Health Scenario; Faces Condition

Question type	Allergy drug/ characteristics	Picture (Happy)	Picture (Neutral)	Picture (Angry)
	Moribol, doctor recommendation	Rating	Rating	Rating
helpful		0-6	0-6	0-6
trustworthy		0-6	0-6	0-6
informational value		0-6	0-6	0-6
	Moribol, usability	Rating	Rating	Rating
helpful		0-6	0-6	0-6
trustworthy		0-6	0-6	0-6
informational value		0-6	0-6	0-6
	Ebonal, prescription	Rating	Rating	Rating
helpful		0-6	0-6	0-6
trustworthy		0-6	0-6	0-6
informational value		0-6	0-6	0-6
	Ebonal, long term efficacy	Rating	Rating	Rating
helpful		0-6	0-6	0-6
trustworthy		0-6	0-6	0-6
informational value		0-6	0-6	0-6

	Zarteg, rate of action	Rating	Rating	Rating
helpful		0-6	0-6	0-6
trustworthy		0-6	0-6	0-6
informational value		0-6	0-6	0-6

	Zarteg, side effects	Rating	Rating	Rating
helpful		0-6	0-6	0-6
trustworthy		0-6	0-6	0-6
informational value		0-6	0-6	0-6

Vacation Scenario; Faces Condition

Question type	Destination/ characteristic	Picture (Happy)	Picture (Neutral)	Picture (Angry)
	Barbuda, landscape	Rating	Rating	Rating
helpful		0-6	0-6	0-6
trustworthy		0-6	0-6	0-6
informational value		0-6	0-6	0-6
	Barbuda, beach	Rating	Rating	Rating
helpful		0-6	0-6	0-6
trustworthy		0-6	0-6	0-6
informational value		0-6	0-6	0-6
	Bonaire, food	Rating	Rating	Rating
helpful		0-6	0-6	0-6
trustworthy		0-6	0-6	0-6
informational value		0-6	0-6	0-6
	Bonaire, service	Rating	Rating	Rating
helpful		0-6	0-6	0-6
trustworthy		0-6	0-6	0-6
informational value		0-6	0-6	0-6
	Caicos, weather	Rating	Rating	Rating
helpful		0-6	0-6	0-6
trustworthy		0-6	0-6	0-6
informational value		0-6	0-6	0-6

	Caicos, costs	Rating	Rating	Rating
helpful		0-6	0-6	0-6
trustworthy		0-6	0-6	0-6
informational value		0-6	0-6	0-6
